# Digital health information on surgical treatment options for overactive bladder is underrepresented

**DOI:** 10.1007/s00345-023-04447-3

**Published:** 2023-06-05

**Authors:** Tanja Hüsch, Sita Ober, Axel Haferkamp, Laila Schneidewind, Matthias Saar, Jennifer Kranz

**Affiliations:** 1 Department of Urology and Pediatric Urology, University Medical Center of Johannes-Gutenberg University, Langenbeckstr. 1, 55131 Mainz, Germany; 2 Department of Obstetrics and Gynecology, Hospital Darmstadt, Darmstadt, Germany; 3 Department of Urology, University Medical Center Rostock, Rostock, Germany; 4 Department of Urology and Pediatric Urology, University Hospital RWTH Aachen, Aachen, Germany; 5 Department of Urology and Kidney Transplantation, Martin-Luther-University, Halle (Saale), Germany

**Keywords:** Social media, Search engine, Overactive bladder, Communications media, Surgery

## Abstract

**Purpose:**

Digital health information gains growing importance in the medical landscape. Despite its opportunities, there is a risk of patient misinformation which may adversely influence the patient–physician relationship. This investigation aimed to identify and compare differences in the content and quality of online health information on overactive bladder (OAB) between different digital platforms.

**Methods:**

The platforms Google search, Facebook, Instagram, LinkedIn, and YouTube were searched for the keyword OAB. The search result links were classified as useful or misleading, advertisement and personal experience. Information regarding the organization of the source and available content on treatment modalities was collected. Descriptive analysis was applied. Univariate and multivariate analyses were performed to evaluate heterogeneity regarding the distribution of information depending on the source. A *p* value < 0.0*5* was considered statistically significant.

**Results:**

The source with the highest quantity of useful content was YouTube (100%) and Google (100%), whereas LinkedIn included mostly misleading content (73%). YouTube and Google provided the greatest variety of health information and were dominated by professional associations. Surgical procedures for treating OAB were only described in 32% and 48% of Google and YouTube results, respectively. On Google, sacral neuromodulation and OnabotulinumtoxinA were described in 26% and bladder augmentation in only 16% of the search results. In contrast, alternative medicine was present in 76%.

**Conclusions:**

A large gap in the information on surgical treatments of OAB could be identified independently from the utilized source. In contrast, conservative treatments and alternative medicine dominate the current informational sources.

**Supplementary Information:**

The online version contains supplementary material available at 10.1007/s00345-023-04447-3.

## Introduction

The digitalization of society is a progressive phenomenon that has increasingly gained irreplaceable importance [1]. Likewise, medicine is undergoing a transformation which can substantially impact the patient–physician relationship based on the availability of health information [2, 3]. However, the success of therapy mainly depends on the patient's cooperation, which can be significantly improved by profound information [4].

Every day, approximately 3.2 billion people—or about 42% of the current global population—spend an average of 1.5 h only on social media [4]. Thus, social media are expected to form the modern medical landscape and will play a pivotal role in accessing health information [5]. Shameful topics, such as urinary incontinence, offer the unique opportunity to search for and communicate health information anonymously [6].

It was estimated that approximately 969 million individuals in 2018 were affected by urinary incontinence and overactive bladder [7], which, subsequently, may seek online health information.

However, the quality of online health information varies and patients need more abilities to assess these information critically [3]. This carries the risk of misinformation, distress, increasing tendency for self-diagnosis or self-treatment, and may even adversely affect the patient–physician relationship [8].

This investigation aimed to identify differences in the quality and content of online health information depending on the utilized source. For this purpose, the online resources Google search, LinkedIn, YouTube, Facebook, and Instagram have been searched for health information about overactive bladder.

## Materials and methods

The platforms Google search, Facebook, LinkedIn, Instagram, and YouTube were searched between March and June 2021 for the keyword “overactive bladder”. The web browser's cache and cookies were deleted before the search, and the search was performed in incognito mode.

The first 30 Google search results, presented in three search engine result pages (SERP), have been utilized for analysis. This is because about 70% of searchers do not pass the first SERP, including 10 search results, and even 67.6% do not even pass the first five results of the first SERP. The click-through rate in the second and third SERP is only 5.59%, emphasizing the relevance of the listing in the first SERP [9]. Therefore, the current search has been limited to the first three SERPs, including 30 search results that have been applied to all platforms [10]. Sponsored search results have been excluded to provide a homogenous result based on Google search rules.

First, the results have been classified into useful [11, 12], misleading, advertising, and personal experience. Useful was defined if the content included scientifically accurate information about any aspect of the disease, including but not limited to prevention, symptoms, treatment, or pathogenesis. On the contrary, misleading content did not include any information about the target disease, such as advertisements, jokes, or job vacancies. Advertising content includes advertisements for specific industry products, whereas personal experiences describe one’s own/subjective experience. Subsequently, the information was categorized by the website’s organization into individual health care professionals (HCP), professional associations (i.e., medical schools, guideline committees, hospitals, etc.), industry, patients, and individuals. The categorization was independently performed by two physicians using the given definitions. Discrepant judgments were discussed with another urologist until consent was reached. The medical content was analyzed and categorized into pathophysiology, diagnosis, and therapy if applicable. Any treatment options presented on the websites were collected.

The readability score, Alexa Score, and Health on the Net foundation evaluation were analyzed in Google search analytics. The Alexa Score is a global ranking system that sorts websites by popularity. It is calculated based on the estimated average daily number of visitors and page views for a given website in the last three months [13]. The lower the Alexa Rank, the more popular the website. An Alexa rank of 1 million or less is considered to be good.

The Flesch–Kincaid Grade level for readability calculates the effort for reading the text for different levels of education. The higher the score, the more challenging is to read the text. The scale ranges from 0 to 18, where 15–18 represents the level of a scientific paper and 0 to 3 learning to read books. Text intended for public readership should aim for a grade level of 8, schooling age 13–14 [14].

The Health on the Net (HON) foundation is a non-governmental organization aiming to ensure quality health information on the internet. HON-qualified internet websites promise verified medical accuracy or correctness and receive a HON code seal if validated [15].

### Statistics

Descriptive analysis has been applied. The Chi^2^ test has been utilized for identifying heterogeneities between the groups. A multivariate logistic regression analysis was performed to identify predictors for specific outcomes. A *p* value < 0.05 has been considered significant. Statistical analysis was performed by SPSS 26 (IBM, Armonk, United States).

## Results

**Fig. 1:**
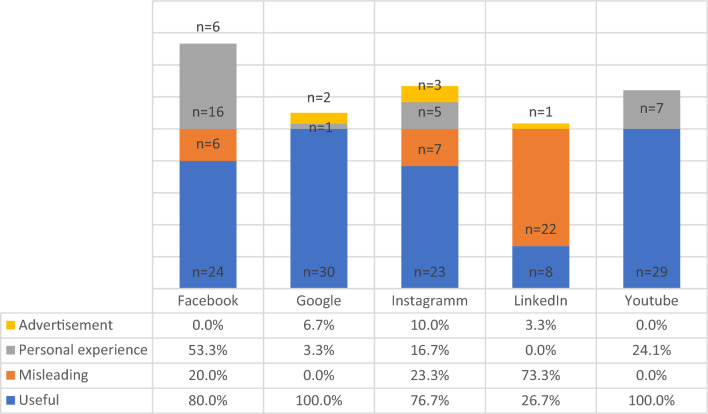
Content classification in comparison between the platforms

There were significant differences in the availability of informative content (*p* < 0.001), personal experience (*p* < 0.001), and advertisement (*p* < 0.001) between the platforms. Google and YouTube had the highest percentage of informative content, whereas LinkedIn content was mainly misleading (Fig. 1). The organizations providing health information varied significantly between the platforms (*p* < 0.001). Professional organizations provided most of their content on YouTube and Google, whereas healthcare professionals provided main content on LinkedIn and Instagram (online resource 1). Professional organizations and healthcare professionals provided the most informative content (92% and 72.1%, respectively). Independent predictors for informative content were professional organizations [*p* < 0.001; OR 7.89 (95% CI 2.51–24.81], whereas independent predictors for misleading information were content provided by individuals [*p* = 0.030; OR 0.25 (95% CI 0.07–0.88)] and industry [*p* = 0.002; OR 0.12 (95% CI 0.03–0.46)].

The target populations of OAB content were mainly non-specific in all platforms. The second largest target population were women in 0–51.7%. Pregnant women were only targeted on Instagram [*n* = 5 (16.7%)], and any source only sporadically targeted other groups, such as men, children, or disabled persons. A HON certificate was available in 13 (43.3%) Google search sites. The mean Alexa score was 328,007, and the median readability score was 9.7.

**Fig. 2:**
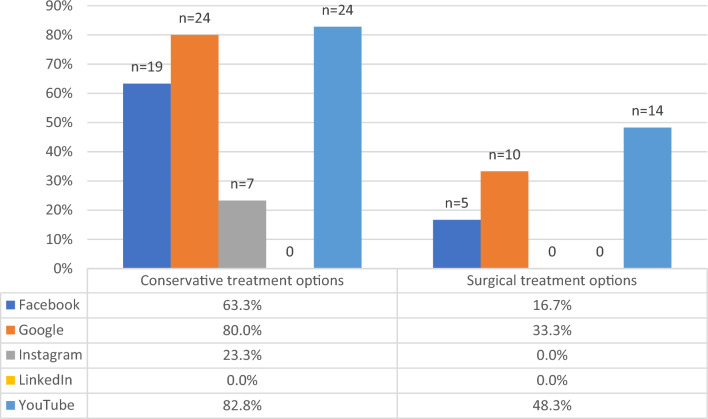
Conservative and surgical treatment content compared between the platforms

Conservative and surgical treatment options were described overall by 74 (49.8%) and 29 (19.5%) hits, respectively, irrespective of the source. Google and YouTube provided the majority of content regarding conservative treatment options (Fig. 2). Homeopathy and alternative treatments were the contents mostly available on Google search in up to 76.7% of all sites. In contrast, surgical treatment options were not described in more than half of all contents. Google provided available surgical treatment of OAB in 33.3% and YouTube in 48.3% (Table 1).

**Table 1:** Pharmacotherapy and surgical treatment options described in the content compared between the platforms

Source	Homeopathy and alternative treatments	Pharmacotherapy	Electrostimulation*	Sacral neurostimulation	OnabotulinumtoxinA	Bladder augmenttation	Urinary diversion
Facebook, *n* (%)	7 (23.3)	14 (46.7)	0	3 (10)	1 (3.3)	0	0
Google, *n* (%)	23 (76.7)	20 (66.7)	9 (30)	8 (26.7)	8 (26.7)	5 (16.7)	3 (10)
Instagram, *n* (%)	4 (13.3)	1 (3.3)	0	7 (23.3)	0	0	0
LinkedIn, n (%)	0	0	0	0	0	0	0
YouTube, *n* (%)	16 (55.2)	17 (58.6)	8 (27.6)	8 (27.6)	12 (41.4)	2 (6.9)	1 (3.4)

*Trans- or percutaneous (e.g., tibial nerve stimulation) or intra-vesical electrostimulation

Pharmacotherapy was described in 67% and 59% of the sites on Google and YouTube, respectively. However, information regarding sacral neuro-stimulation and OnabotulinumtoxinA was found in only 26.7% of the sites on Google, respectively, and 27.6% and 41.4% on YouTube sites, respectively. Bladder augmentation and urinary diversion were reported in up to 16.7% of the contents or missing entirely (Table 1).

## Discussion

This investigation aimed to evaluate the content of digitally available online health information on overactive bladder compared to different digital platforms. We identified that overactive bladder is a popular search term on Google search, and a HON seal qualifies about 43% of webpages. The platforms with the most considerable informative content were found on Google and YouTube, targeting predominantly unspecific and female populations. LinkedIn was identified associated with the source of the highest amount of misleading content dominated by industry and healthcare professionals. Considering medical content, Google, YouTube, and Facebook provided information regarding conservative treatment options in most searches. However, conservative treatment options using homeopathy and other alternative regimes dominated the conservative content, even over pharmacological treatments. The overall available information about surgical treatment options of OAB, irrespective of the source, was only 19.5%. When focusing on Google and YouTube, this number increased only to 33% and 48%, respectively. The information on standard procedures such as OnabotulinumtoxinA injections or sacral neuromodulation was mentioned only in one of four Google searches. More invasive procedures, such as bladder augmentation or urinary diversion, were sparse.

Unsurprisingly, overactive bladder is a popular search term identified by a low Alexa rank. Urinary incontinence is of high prevalence, mainly reported between 35 and 45% [16]. Yet, it is still considered taboo and few seek treatment [17]. Digital resources offer the possibility to seek anonymous information on shame-faced topics [6]. Although the information found in digital resources by patients may positively affect patients´ compliance and treatment, there is a risk of misinformation due to the inhomogeneous quality of the content, which may lead in contrast to fundamental damage to the physician–patient relationship and harmful self-treatments [3, 8].

A possible source of misinformation is the quality and completeness of the information and the readability. According to the Flesch–Kincaid Grading, the median readability score in this investigation was 10, which may be difficult for the average reader to understand. The score should target a Grading of 8 to ensure an understanding of the content [14].

More emergently is the lack of proper information regarding pharmacological and surgical treatment options for overactive bladder. Up to 67% of the content informed about pharmacotherapy, and only 20% of the overall content included any surgical options at all. Surprisingly, even in a Google search, the standard treatment options with OnabotulinumtoxinA injections and sacral neuromodulations were present on only one of four websites. Information on bladder augmentations or urinary diversion was even more sparse, which reported only in up to 15%. Thus, this evaluation first identified a large gap in adequate digital health information for the overactive bladder surgical treatment options. Since urinary incontinence is a shame-faced topic, missing information on adequate surgical treatment options may adversely affect the patients by discouraging patients seeking medical support. In contrast, the information on homeopathic and/or alternative medicine treatments was available in up to 77%. There is limited evidence on phyto-therapy used for overactive bladder [18]. However, the robustness of the clinical trials is often limited, and individualized herbal recipes are often used, which limits the generalization of the results [19, 20]. Although alternative treatment options should be considered when informing patients, the standard treatment of care should be the reference for any consultations.

The availability of informative content on overactive bladder differed significantly. Google and YouTube have been identified as the sources with the most informative content. In contrast, LinkedIn was identified to provide majorly misleading content and very little information about treatment options. This is in line with other findings on benign pelvic diseases with comparable results for the search term pelvic organ prolapse [21]. Industry-sponsored content and content from individuals were identified as independent predictors for the reduced likelihood of informative digital health information. This may be referred to limited individual knowledge of the disease and industry content is often referred to job descriptions or advertisements, which limits the value of the content.

The majority of the target population was unspecified and women. Overactive bladder is the most common symptom in neurourological patients, with prevalence up to 71% and 96% in patients with Morbus Parkinson or multiple sclerosis, respectively. However, other diseases, such as dementia, neuropathy due to diabetes mellitus, cerebrovascular diseases, and spinal cord injuries, are commonly associated with OAB [22]. In addition, the prevalence of OAB in children is between 5 and 12% [23]. Neurourological patients and children are vulnerable populations with specific requirements which are not adequately addressed by the current digital health information.

Finally, certified HON sites were available in 43% of the results. The availability of the seal in other medical diseases has been reported between 1.8 and 42.9% [24–26]. Thus, the current results are located at the upper end of the reported availabilities, which may be referred to a more widespread use of the HON seal over the years. However, the high availability of the HON seal contradicts the limited information on the surgical treatment of OAB. This questions the robustness of the HON seal if substantial information on a disease treatment is not included in a certified website.

We acknowledge a few limitations of this investigation. The searches were limited to a total of 30 hits for each platform. However, as previously described, the majority of users will not consider any results even after the first 10 findings which emphasized the relevance of this investigation. Furthermore, not all digital platforms have been utilized and there might be other platforms providing different results. However, this investigation provides a comprehensive overview of the most used digital platforms worldwide on the search term overactive bladder. It identifies crucial limitations of current digital health information.

## Conclusions

Overactive bladder is a popular search term for patients seeking digital health information on digital platforms. Google and YouTube provide the most informative content, whereas LinkedIn is dominated by misleading information and lacks content on OAB treatment. The target populations are unspecified and women, excluding hereby vulnerable populations, such as neurourological patients and children. Although conservative treatments were available in most results, they were dominated by homoeopathic and alternative medicine results. Furthermore, a large gap in informative content on surgical treatment options of OAB has been identified for the standard of care procedures. Only one out of four Google searches provided information on OnabotulinumtoxinA or sacral neuromodulation, which has yet to be completely missing on other platforms. There is an emerging need for adequate digital health information on treatment options for patients seeking online information on OAB.

These results highlight the emerging role of the urological and gynecological community in providing objective, up-to-date, complete, and correct information in digital channels. However, taking responsibility for digital medical information content is only one pillar of successful education. The complex algorithm of digital platforms requires a close collaboration with professionals in informational technology to lead adequate medical education in a broad spectrum to success.

## Supplementary Information

Below is the link to the electronic supplementary material.

Supplementary file1 (DOCX 28 KB)

## Data Availability

Research data are not shared.
