# Supplementary material for: Digital health information on surgical treatment options for overactive bladder is underrepresented
Source: World J Urol. 2023 Jun 5;41(7):1891–6. doi: 10.1007/s00345-023-04447-3 (PMC10352412; doi:10.1007/s00345-023-04447-3)
Supplement: Supplementary file 1 — Supplementary file1 (DOCX 28 KB) [file 345_2023_4447_MOESM1_ESM.docx]

Figure 2 Organizations providing content in comparison between the platforms
